# Estimating public health risks of infectious disease events: A Canadian approach to rapid risk assessment

**DOI:** 10.14745/ccdr.v50i09a01

**Published:** 2024-09-05

**Authors:** Sai Priya Anand, Clarence C Tam, Sharon Calvin, Dima Ayache, Lisa Slywchuk, Irene Lambraki, Rukshanda Ahmad, Jan Trumble Waddell, Eleni Galanis, Linda Vrbova

**Affiliations:** 1 Centre for Surveillance, Integrated Insights and Risk Assessment (SIIRA), Data, Surveillance and Foresight Branch (DSFB), Public Health Agency of Canada, Ottawa, ON; 2 School of Population and Public Health, University of British Columbia, Vancouver, BC; 3 School of Epidemiology and Public Health, University of Ottawa, Ottawa, ON

**Keywords:** risk assessment, qualitative rapid risk assessment, risk pathway, infectious disease event, public health threats

## Abstract

**Background:**

The COVID-19 pandemic highlighted the need for timely, evidence-based rapid risk assessments (RRA) of infectious disease events to inform public health action during rapidly evolving situations with high uncertainty. In 2022, the Public Health Agency of Canada established a coordinated approach to public health risk assessment, including a methodology for qualitative RRA of infectious disease threats.

**Objective:**

To describe the RRA methodology and illustrate its use with examples from different infectious hazards of public health concern.

**Methods:**

The RRA methodology employs the risk pathway to describe the sequence of events leading from a hazard’s source to the adverse event of concern and subsequent impacts; define specific questions to be addressed; and identify relevant knowledge gaps, limitations and recommendations. Qualitative likelihood and impact estimates are derived through integration of evidence review and expert opinion and are communicated together with corresponding levels of uncertainty. The impacts of the event are based on an assessment of the most likely spread scenario within Canada, considering individual-level impact on affected individuals, the impact on the general population and, if relevant, sub-groups at higher risk.

**Results:**

This RRA approach aligns with well-established international methods and provides flexibility to accommodate a broad range of risk questions. It has been implemented to estimate the risk of various threats of concern to Canada, including mpox, avian influenza A(H5N1) and measles.

**Conclusion:**

Given the broad range and complexity of public health hazards, RRAs provide a timely, coordinated and systematic process for characterizing and communicating the risk to inform risk mitigation and decision-making and to guide appropriate public health response.

## Introduction

The COVID-19 pandemic highlighted the need for a risk- and evidence-based approach to implementing public health measures in the context of a rapidly evolving situation with limited information and high uncertainty. Rapid risk assessments (RRAs) provide a systematic approach to gathering, assessing and documenting information about a public health hazard, to assign a level of risk to inform decision-making within a short timeframe (1,2). Rapid risk assessments are, therefore, crucial in the early response to a public health event as they provide risk managers with a timely and evidence-based assessment of the risk and associated levels of uncertainty upon which to base risk management, surveillance and research recommendations (3,4). Additionally, RRAs can be updated, taking into account new information as an event evolves.

Based on recommendations from the Auditor General’s Report on Pandemic Preparedness, Surveillance, and Border Control Measures (5) and the Global Public Health Intelligence Network Independent Review Panel (6), in 2022, the Public Health Agency of Canada (PHAC) consolidated risk assessment activities across PHAC to establish and coordinate an integrated risk assessment approach to RRA. The RRA process is initiated when a threat is identified (e.g., through signal detection or surveillance) for which an estimation of the associated risk is needed to inform public health preparedness and response. Due to the need for timely response and the limited information available during the early stages of an event, RRAs are typically qualitative in nature, involving a combination of evidence review and expert opinion. In this paper, we describe the development and methodology of PHAC’s qualitative RRA approach and illustrate its use with different infectious hazards of public health concern as examples.

## Rapid risk assessment methodology

### Development

Four qualitative public health RRA approaches (1,2,7,8) were initially identified through an informal environmental scan of risk assessment approaches utilized by international public health organizations (e.g., UK Health Security Agency, European Centre for Disease Prevention [ECDC] and Control and World Health Organization [WHO]) as well as peer-reviewed publications, grey literature and expert input of threat and risk assessment frameworks and methodologies. The four approaches were subsequently tested in the Canadian context using scenarios and historical infectious disease events. Two approaches use an algorithm to determine the risk level posed by infectious disease events ((1,7)), while the other two involve development of specific questions to be addressed related to the likelihood and impact of the event of concern (2,8). The RRA approach described herein is largely based on the Joint Risk Assessment Operational Tool (JRA OT), developed by the Food and Agriculture Organization (FAO), the World Organization for Animal Health (WOAH) and the WHO ((8)), as a qualitative approach that can be conducted rapidly to inform decision-making for emerging events. The JRA OT was chosen based on its 1) flexibility to accommodate a wide range of risk questions, 2) high level of scientific validity by employing a risk pathway model as a framework to assess likelihood and impact, 3) ability to incorporate One Health considerations in the risk assessment process to address the many hazards that intersect human-animal-plant-ecosystem health and 4) ability to provide sufficient guidance material for implementation and adaptable tools to facilitate the RRA process (e.g., terms of reference for committees). The iterative JRA process at-large has been adapted based on organizational mechanisms and structures in-place and informed by lessons learnt via internal pilot-testing with infectious disease events that occurred in 2022.

### Overall process

When the RRA process is triggered, an event-specific steering committee is formed comprising decision-makers, senior staff in key program areas and relevant external partners. The steering committee’s role is to determine if a RRA is needed, oversee the RRA process, define the scope and key objectives of the assessment, review findings and recommendations and communicate these to relevant decision-makers. In the execution phase, a multidisciplinary technical team comprising risk assessors and subject matter experts (SMEs) conducts the assessment by mapping a risk pathway; finalizing the risk question(s) to be addressed; gathering and synthesizing evidence; assigning likelihood, impact and uncertainty levels; identifying assumptions, limitations and knowledge gaps; and providing recommendations for risk mitigation, surveillance and research. These are summarized in a report that includes an overall risk statement and recommendations for risk management that are approved and communicated to relevant stakeholders in the dissemination phase (**Box 1**).

Box 1 **Overall process for conducting a public health rapid risk assessment for infectious disease events^a^**

1. Initiation phase: setting the stage

• Establishing a steering committee and technical team

• Risk framing and formulating risk question(s)

2. Execution phase: conducting the assessment

• Diagramming risk pathway(s)

• Finalizing risk question(s) and formulating pathway sub-questions

• Gathering and synthesizing evidence

• Assigning likelihood, impact and uncertainty levels

• Identifying assumptions, limitations and knowledge gaps

• Formulating risk statement and risk management recommendations

3. Closure phase: recommendations, communication and update

• Reviewing and approving RRA findings and recommendations

• Disseminating RRA outcomes and report

• Developing triggers for re-assessment of risk

• Monitoring emerging evidence and situational assessment

• Updating the assessment as the event evolves

Abbreviation: RRA, rapid risk assessment^a^ This process is situation-dependent and not linear; it is often adapted to the hazard, scope, purpose and timelines, as established during risk framing

## Initiation phase: setting the stage

### Risk framing

The steering committee conducts a risk framing (problem formulation) exercise to determine whether a RRA is needed and outline the scope and key objectives of the assessment. This includes defining the following:

• The public health hazard (pathogen or other threat) that poses a potential risk

• The public health concerns related to the hazard

• The adverse event of concern (the event to be avoided or mitigated); e.g., introduction of an infectious individual with disease X into Canada

• The source of the hazard

• The at-risk population(s) of interest

• The timeframe over which the risk should be assessed

• The contextual factors that can influence the likelihood or impact of the event; e.g., conditions affecting exposure or transmission, available countermeasures and resources for risk mitigation

• The relevant stakeholders (including those whose expertise is required to conduct the assessment and those to whom the results of the assessment should be communicated)

• The risk management decisions that should be informed by the RRA; e.g., border health measures, infection prevention and control guidance

This risk framing aids in the formulation of the specific risk question(s) to be answered during the RRA (see **Table 1** for more examples).

**Table 1:** Risk framing leading to the risk question for assessment for different infectious disease agents of public health concern

Hazard	Adverse event of concern	Source population(s)	At-risk populations	Timeframe	Risk question
VHF disease outbreak in Country X	Introduction of infected human into Canada	Immigrants resettling to Canada, travellers including tourists, Canadians visiting home countries or on business trips	Close contacts of infected individual, general population	4 weeks	What is the likelihood and impact of a VHF disease introduction into Canada from the outbreak in Country X within the next four weeks?
Avian influenza A(H5N1) clade 2.3.4.4b virus (9)	Human infection in Canada	Wild birds, domestic birds, wild mammals, domestic mammals	Individuals with higher-level exposure^a^, individuals with lower-level exposure, general population	Current and up to the end of the next bird migratory season in Canada	What is the likelihood and impact of at least one human infection with avian influenza A(H5N1) clade 2.3.4.4b due to exposure to either birds or mammals in Canada up to the end of the 2023 fall bird migratory season?
Poliovirus outbreak in Country X	Infection of an un/under-vaccinated person in Canada	Immigrants resettling to Canada, travellers including tourists, Canadians visiting home countries or on business trips	Un/under-vaccinated close contacts, un/under-vaccinated communities	4 months	What is the likelihood and impact of poliovirus importation and transmission to un/under-vaccinated close contacts in Canada associated with the poliovirus outbreak in Country X within the next four months?
2022 global mpox outbreak (10)	Human-to-human transmission in Canada	Travellers to Canada from endemic regions at the start of the outbreak, limited clusters of domestic transmission	gbMSM, trans and gender-diverse people, sex workers in Canada, individuals with multiple sexual partners and their close contacts, general population	4 weeks	What is the likelihood and impact of mpox virus transmission among gbMSM with multiple sexual partners and their close contacts in Canada within the next month?

Abbreviations: gbMSM, gay, bisexual, and other men who have sex with men; VHF, viral hemorrhagic fever

^a^ High intensity contact (within two meters and/or prolonged without use of personal protective equipment) with animals infected with avian influenza A(H5N1) clade 2.3.4.4b virus (i.e., wild birds, poultry or mammals), infected materials from these animals (e.g., feces, blood, secretions or tissues) or an environment highly contaminated by infected animals

## Execution phase: conducting the assessment

### Risk pathway

**Figure 1:**
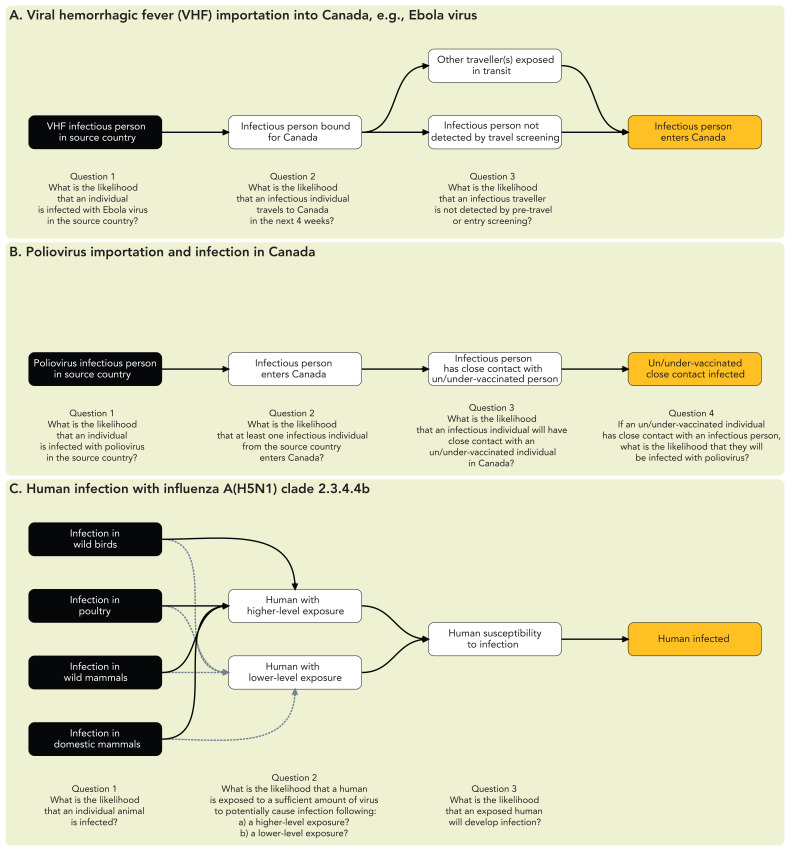
Risk pathway diagrams depicting the steps from the source of infection to the adverse event of concern

The risk framing informs the development of a risk pathway: a diagram describing the sequence of events leading from the hazard’s source to the adverse event of concern and its resultant impacts. Each box (node) in the diagram represents a step along the risk pathway; arrows (edges) depict causal relationships, linking each event to its consequences (**Figure 1**). The likelihood of a specific event occurring is, therefore, conditional on preceding events. Typically, a risk pathway for an infectious disease hazard includes components describing the importation, if relevant, of the hazard (pathogen) from the source country, exposure to the hazard within Canada, human infection, the most likely spread scenario should infection occur and the resulting impacts. The adverse event of concern (the event to be avoided or mitigated) should be clearly defined, since this is the event for which the overall likelihood will be assessed. Other components such as potential interventions (e.g., vaccination, treatment) and monitoring points (e.g., surveillance systems) can be added to the pathway where relevant. Mapping out the risk pathway helps to formulate the risk question that is of key concern and the types of information that will be needed to address it.

Risk pathways can vary in complexity depending on the hazard and risk question and individual components of the pathway can be expanded and assessed in greater detail or simplified as needed. For example, in considering the potential for importation of a non-endemic disease into Canada (e.g., Ebola virus) into Canada, measures such as pre-travel health screening could reduce the likelihood of importation by preventing symptomatic individuals in the source country from travelling (Figure 1A). With infectious diseases that mostly present as asymptomatic (e.g., poliovirus), such measures are unlikely to meaningfully influence the likelihood of importation; thus, the importation component of the risk pathway can be simplified, assuming that pre-travel health screening would not detect poliovirus infections (Figure 1B). The level of detail in a risk pathway are dependent on the time and resources, availability of information, complexity of the risk questions, risk management needs and sensitivity of the risk to specific steps in the pathway.

Similarly, the risk pathway approach allows for flexibility in incorporating components at the human-animal-ecosystem interface. For example, when assessing the risk of human infection with influenza A(H5N1) clade 2.3.4.4b viruses, the risk pathway can include the likelihood of infection in relevant animal species and the likelihood of human exposure and infection (Figure 1C); depending on the scope, the assessment could consider impacts on human health, the economy, wildlife and agriculture. Risk pathways are, therefore, useful for incorporating multi-sectoral perspectives within a One Health approach.

The risk pathway is used to develop specific sub-questions to be answered during the risk assessment. These sub-questions correspond to individual steps (nodes) in the pathway influencing the likelihood of the event of concern (likelihood sub-questions) and steps leading from the event of concern to the impacts being assessed (impact sub-questions). For infectious hazards, assessing the impacts typically requires an assessment of the most likely spread scenario(s) should the event of concern occur. It should be noted that the event of concern can differ depending on the context and the specific objectives of the assessment (Figure 1, orange nodes).

**Figure 2:**
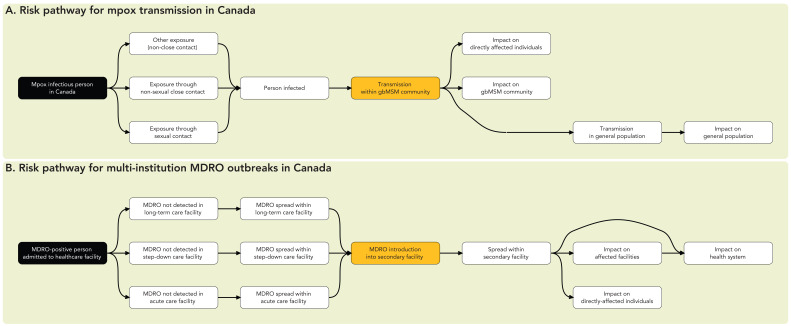
Risk pathways for mpox and multidrug-resistant organism transmission Abbreviations: gbMSM, gay, bisexual, and other men who have sex with men; MDRO, multidrug-resistant organism

Risk pathways can be adapted to consider at-risk populations or settings within the RRA (**Figure 2**). For example, the risk pathway can capture the likelihood and impact of infection in defined sub-populations of concern, such as specific occupational, demographic or other relevant high-risk groups (Figure 2A). A further consideration is that the unit of analysis may differ depending on the context. Figure 1 and Figure 2A depict risk pathways related to the likelihood of an individual importing, transmitting or acquiring a pathogen. Figure 2B depicts a risk pathway in which the event of concern is the spread of a multidrug-resistant organism between healthcare facilities, making a healthcare facility a more appropriate unit of analysis.

### Estimation of likelihood and impact

Once the risk pathway is complete, evidence to address each pathway sub-question is compiled, reviewed and appraised to produce qualitative estimates of likelihood and/or impact together with levels of uncertainty (see more on uncertainty below). Estimates are informed by a rapid review of relevant evidence, which can include scientific literature, published and unpublished technical reports and epidemiological investigations, event and case-based surveillance data, intelligence obtained through international networks and reporting systems and scientific expertise. As time and evidence can be limited during the early stages of an event, RRAs rely on expert knowledge and opinion. Subject matter experts in relevant areas can guide the estimation process by providing contextual or privileged information about the hazard being assessed, a nuanced interpretation of the evidence and expert judgement on the event of interest and surrounding context, such as relevant sociocultural factors and industry practices. Uncertainty is estimated based on the availability and quality of relevant evidence, SME opinion and degree of expert agreement. For each pathway sub-question, a qualitative estimate of the likelihood or impact is assigned using pre-defined, standardized scales describing how likely an event is to occur and what impact it is expected to have, both among directly affected individuals and the wider population. Each estimate is accompanied by a brief, focused rationale summarizing the evidence that supports the level assigned. Scales for likelihood and impact estimation available in existing risk assessment frameworks (2,8) can be adapted to suit the local and situational context (see RRAs of measles in Canada and influenza A(H5N1) clade 2.3.4.4b virus for current likelihood, magnitude of effect, impact and uncertainty scales (11,12)).

### Likelihood considerations

**Figure 3:**
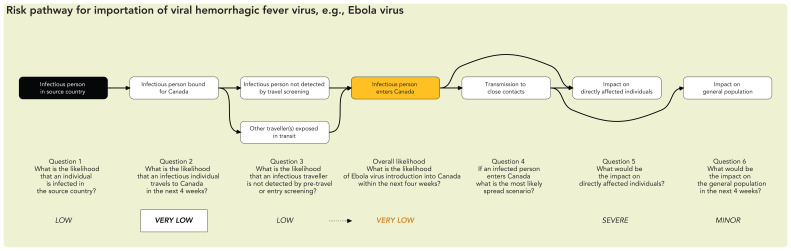
Risk pathway for the importation of a viral hemorrhagic fever disease

The overall likelihood is a qualitative statement of probability that the adverse event of concern will occur within the time period of interest. The overall likelihood for the adverse event is conditional on the likelihood estimates for preceding steps in the risk pathway and is derived in a manner analogous to the quantitative multiplication of probabilities. When multiplying conditional probabilities, the overall probability can never be higher than the lowest individual probability in the pathway. In the qualitative equivalent, the overall likelihood should not be higher than (and is thus determined by) the lowest likelihood estimate in the pathway (**Figure 3**) (13).

Considerations for assessing the likelihood of an infectious disease event depend on the context and the pathway sub-question (examples in **Appendix,** **Table A1**). For example, when considering the likelihood of importation of a disease, relevant factors include the prevalence of infection and epidemic trajectory in the source country, the volume of incoming travellers, health screening measures, the potential for transmission of infection during transit, the case-to-infection ratio and incubation period (which influence the likelihood of infected individuals being detected by surveillance or screening) and the duration of infectiousness (which influences the likelihood of an individual being infectious at the time of travel). Similarly, an assessment of the likelihood of infection in Canada should consider the potential for exposure to infectious individuals, the intensity of exposure, pathogen infectivity and demographic, medical, social and other factors that may influence susceptibility to infection.

### Impact considerations

The estimation of impact conveys the severity of consequences resulting from the adverse event of concern, should it occur. The PHAC RRA approach assesses impacts at the individual and population levels. Impacts on individuals affected by the hazard are informed by evidence regarding disease severity and associated sequelae, availability and efficacy of prophylaxis and treatment and intrusiveness of control measures (e.g., isolation, quarantine). Population-level impacts are additionally dependent on the most likely scenario for the extent and duration of spread.

The most likely spread scenario is influenced by the pathogen transmissibility (e.g., the reproduction number, *R*), speed of transmission (e.g., serial interval, epidemic doubling time) and effectiveness of public health measures (e.g., case detection, case isolation, contact tracing) and medical countermeasures (e.g., antimicrobials, vaccines). It is not necessarily a description of what will happen; in some situations, all spread scenarios may be unlikely to occur if they are contingent upon earlier steps in the risk pathway that are themselves unlikely. Conveying how likely a chosen spread scenario is to occur is useful for providing appropriate context for estimated impacts.

Public Health Agency of Canada’s assessments typically focus on direct health impacts, but also consider additional impacts on wellbeing (e.g., mental health, long-term disability), impacts on the health system, impacts arising from implementation of public health measures or wider impacts using a social, technological, economic, environmental, political and regulatory and population and health system (STEEPP) framework depending on the risk framing (2,14). Balancing these different impacts is challenging and the prioritization of impacts to be assessed should be clarified at the outset, based on the needs of decision-makers and the risk framing exercise. For example, Ebola virus is likely to have severe consequences for infected individuals because of the high case fatality and the need for case isolation but could have minimal population consequences if little onward transmission is expected to occur. Conversely, seasonal influenza is not expected to cause severe illness in most infected individuals but could have major population consequences due to the large number of cases and resultant pressure on the health system.

### Sub-populations with disproportionate impacts

The effects of an event may not be uniformly distributed across the population. Certain population sub-groups may be disproportionately affected due to shared risk factors, occupational exposures, demographics, medical vulnerabilities or socioeconomic circumstances. Infectious disease outbreaks that are highly concentrated in certain sub-groups may have minimal impact on the general population. Assessing only the general population impact may mask significant impacts on specific sub-groups and may have downstream health equity implications. Furthermore, it is important to distinguish between sub-groups that are disproportionately affected because of shared risk factors for infection and sub-groups that are more susceptible to severe consequences of infection, as these may not always be the same groups. For example, during the ongoing global outbreak of mpox, transmission has predominantly occurred among gay, bisexual and other men who have sex with men (15) despite high susceptibility to mpox in the general population. Consequently, the health impacts have overwhelmingly affected this sub-population, while the impact on the general population has been minimal (notwithstanding potential elevated risk of severe outcomes in certain sub-groups such as pregnant women) (16). In contrast, generalized community transmission of SARS-CoV-2 has been the norm throughout the COVID-19 pandemic, but the health impacts have been disproportionately high among the elderly, those with co-morbidities and under-vaccinated individuals, because of their higher susceptibility to severe illness. Assessing the differential impact on specific population sub-groups may, therefore, be necessary for certain hazards and this should be considered in the risk framing, risk pathway and assessment of likelihood, spread and impact.

### Integrating evidence and expert opinion

Evidence used to estimate likelihood and impact is triangulated with expert knowledge to support the overall assessment. Expert opinion is particularly valuable during a RRA when evidence is limited or conflicting, as well as to provide contextual information about the event based on prior experience. Additionally, expert input can help to identify key uncertainties and knowledge gaps in relation to the risk question. The overall timeframe and number of SMEs involved in a RRA can vary depending on the event and the complexity of the issue. For events with less complexity, such as the risk associated with Ebola virus importation, a smaller group of SMEs may suffice and discussion with the aim to build consensus on risk estimates may be feasible. For more complex events, such as the risk associated with human infection with an influenza A(H5N1) clade 2.3.4.4b virus, experts from multiple sectors may be needed, including human and veterinary medicine, public health, virology, immunology, agriculture and environmental science. In such cases, different options can be considered to obtain balanced input from experts. Strategies can vary from requesting targeted input on sections most relevant to experts’ field of knowledge to obtaining initial estimates of risk from experts through surveys ahead of group discussions, to help minimize biases and ensure that all relevant views are represented.

### Levels and drivers of uncertainty and knowledge gaps

For each likelihood and impact level assessed, a level of uncertainty is assigned based on the availability and strength of relevant evidence, as well as expert opinion. As RRAs are typically conducted in the context of limited data, outlining the level of uncertainty for the likelihood or impact of different steps of the risk pathway is crucial for delineating the weight of evidence supporting individual estimates and provides important contextual information for decision-makers to guide appropriate actions (17). In addition to uncertainty levels, identifying drivers of uncertainty and variability is important for determining when actions based on the precautionary principle might be warranted and for defining triggers for re-evaluation of the risk, such as changes in epidemiology. For example, it is not possible to pinpoint which human-adaptive mutations will occur in influenza A(H5) strains within a given timeframe; this is inherently unknowable and will always be highly uncertain (i.e., has high variability). The uncertainty in the likelihood of a viral hemorrhagic fever disease importation, on the other hand, is influenced by availability of information on the extent of transmission, the specific groups in which transmission is occurring, the effectiveness of control measures and the expected volume of inbound travel from the source country. Identifying information gaps can inform surveillance and research recommendations. For example, during an assessment of the risk of human infection with avian influenza A(H5N1) clade 2.3.4.4b virus, gaps identified included lack of evidence regarding the infectious dose in humans and the types of exposures necessary for infection. Consequently, recommended actions included enhancing and integrating surveillance activities for avian influenza across the One Health spectrum in Canada to understand infection risk in human population groups with higher exposure (e.g., agricultural workers) and rapid information sharing of case detections (11).

## Assumptions and limitations

During the assessment, certain assumptions may be necessary to make estimation possible in the face of limited information. For example, data on the frequency of and risk factors for severe illness following human infection with avian influenza A(H5N1) clade 2.3.4.4b virus are currently limited, given the small number of human infections that have been identified to date. Some similarity between this and other influenza A(H5) viruses in the propensity to cause severe illness may, therefore, need to be assumed. Any assumptions made during the assessment are described and any relevant limitations that could influence the outcome or limit the scope of the assessment are listed.

## Closure phase: recommendations, communication and update

### Summary statement and recommendations

The key findings of the RRA are described in a risk statement that summarizes the likelihood and impact estimates, main drivers influencing the estimates and key sources of uncertainty in the assessment. The risk statement, identified knowledge gaps and recommendations form the main outputs of the RRA report. Risk management decisions are outside the scope of RRA and may be based on factors other than assessed risk, including the level of risk tolerance, resource availability, cost-benefit analyses or acceptability of different control measures. However, providing recommended actions helps inform decision-makers, risk managers and relevant stakeholders on risk management options that are proportionate to the risk posed by a given public health hazard. These can include specific actions for response, such as surveillance, implementation of control measures or risk communication to mitigate risk at different levels (e.g., federal, provincial or territorial levels in Canada), as well as research to address knowledge gaps.

If there is considerable uncertainty regarding the likelihood and potential impact of an adverse event beyond the timeframe of the RRA (e.g., for pathogens with pandemic potential or for which the epidemic trajectory is highly uncertain), a description of plausible future scenarios or considerations influencing future risk can be included in the assessment to guide preparedness planning ((11,12)).

### Updating the rapid risk assessment

As a public health event evolves and more information becomes available, a re-assessment of the risk and associated uncertainties may be required to ensure that ongoing risk management activities are appropriate. As part of the PHAC RRA process, monitoring indicators are defined that, if met, would indicate a worsening of the situation and trigger a reassessment of whether an updated RRA is needed. For example, increase in case counts, increased disease severity or case detections in new countries or regions of a given infectious disease outbreak could all trigger a re-evaluation of the risk. In future iterations of an RRA, the risk pathway and risk question(s) may need to be revised if the epidemiological situation changes significantly.

## Discussion

We have demonstrated the application of a coordinated approach to public health RRA in the Canadian context, with a focus on infectious disease events. This method uses the risk pathway as a flexible framework to characterize the likelihood and impact of a public health event of concern, aligned with established international RRA frameworks (2,8). As part of the RRA, estimates of likelihood and impact are reported separately to adequately inform risk management decisions.

Alternative RRA frameworks, such as those used by the ECDC (1) and the UK Human Animal Infections and Risk Surveillance group (7), use algorithms to guide risk assessors through a pre-determined decision process to derive estimates of likelihood and impact. Algorithm approaches have the advantage of using a standardized set of questions for every risk assessment and of being intuitively easier to understand for both risk assessors and decision-makers. In our experience, however, the risk pathway approach provides greater flexibility than a binary decision process when a more nuanced assessment is required and this approach may be easier to adapt for a broad range of hazards. This is primarily made possible with the ability to craft risk questions specific to the public health event being assessed to ensure that the RRA outputs are practical and relevant for the required risk management decisions under consideration by the steering committee.

Ongoing developments to PHAC’s RRA methodology include enhancing approaches for rapid expert elicitation, broadening and improving assessment of impacts beyond health, potentially integrating qualitative and quantitative approaches to inform assessments, strengthening the assessment of quality of evidence, including variability in estimating uncertainty and exploring the possibility of expanding the methodology to hazards other than infectious diseases. Expert judgement, while critical for qualitative RRAs, is known to be prone to various biases (18). These can be mitigated through rigorous elicitation protocols and training of experts in subjective probability judgements, neither of which are easily implemented within the timeframe of a RRA. More work in rapid expert elicitation is required to develop flexible protocols and training material that can be implemented during RRAs.

Although the human health impacts of a public health event may be of primary concern in many risk assessments, in some situations, the economic and social impacts may be substantial. Examples include the economic impacts of highly pathogenic avian influenza on the agricultural sector and the wide-ranging impacts of the COVID-19 pandemic. Commonly used qualitative impact scales, such as that recommended in the JRA OT (8), incorporate some of these dimensions, but can be difficult to use in practice because they require judgements about the relative importance of health and other impacts. The development of separate scales to capture impacts in different domains, such as impacts on health, the health system, the environment and wider society, could provide a more specific characterization of the types of impacts expected from both infectious and non-infectious hazards (14,19).

Increasingly, developments in mathematical modelling are being used to provide simulations and timely forecasts of likelihood of importation, epidemic spread and the impact of control measures to aid decision-making. Exploring how to integrate quantitative approaches into RRAs may help provide additional understanding of the potential impacts of an event, the key factors influencing those impacts and what public health actions should be prioritized to minimize impacts.

Risk assessment, an evolving field within public health, is important for informing timely and evidence-based decision-making. Given the broad range and complexity of public health hazards, RRA provides a coordinated approach to characterizing and communicating the level of risk to public health posed by an event of concern, to help prioritize and inform risk management activities. When the public health risks fall at the intersection of human-animal-plant-ecosystem health, a multisectoral One Health approach to risk assessment can reduce duplication of effort, improve timely sharing of information across sectors, enhance focus on upstream drivers of health risks and impacts across sectors and facilitate engagement of multiple sectors in risk management measures.

## Appendix

**Table A1:** Examples of relevant considerations for different risk pathway steps and types of evidence and uncertainties informing the assessment

Example risk pathway steps	Example considerations	Types of evidence informing assessment	Uncertainty considerations
Infection in source country	What is the epidemiological situation? What is the geographic distribution of disease? Are specific population sub-groups being affected?	Case reports, event summaries, surveillance data, local and international risk assessments, intelligence from relevant agencies/organizations	Extent of under-ascertainment of cases, role of asymptomatic and pre-clinical infections in transmission, delays in reporting of cases and deaths through surveillance mechanisms, potential biases in case detection towards certain geographic regions (e.g., urban areas) or population sub-groups (e.g., children)
	How will the epidemic evolve over the period of assessment?	Information on the extent, speed and potential for epidemic spread; epidemic doubling time; evidence for widening of geographic range or population groups affected; immunization coverage (for vaccine-preventable diseases); effectiveness of public health control measures being implemented	Effectiveness of control measures over the period of assessment, changes in epidemiology and/or pathogen biology
Importation from source country	What is the expected volume of travel from the source country over the period of assessment?	Data on forecasted air passenger travel volumes over the period of assessment	Historical trends may not reflect travel patterns during period of assessment Limited information or travel by other routes (e.g., land, sea)
	Are potentially infectious individuals likely to travel to Canada?	Quantitative models of importation risk	Importation models may not accurately capture travel by specific population sub-groups of interest or through non-air travel routes
	Are infectious travellers likely to infect others during transit?	Epidemiological, microbiological and environmental studies and risk assessments of transmission risk during transit	The frequency and duration of potentially infectious exposure events during transit is likely to be unknown
	Will existing travel health screening and border measures reduce likelihood of importation?	Incubation period, duration of infectiousness, role of asymptomatic and pre-symptomatic infection in transmission, evidence of importation from similar events in the past	Information on what types of travel health screening and border measures are implemented in source, transit and destination countries and their effectiveness, may be limited
Exposure in Canada	What is the size of the population(s) at risk?	Census data, immunization coverage (for vaccine-preventable diseases), information on size of occupational groups, representative survey data on risk factor prevalence, spatio-temporal distribution of competent vectors (for vector-borne pathogens)	Information may not be available on the size and geographic distribution of specific population sub-groups of interest
	What is the frequency and intensity of exposure to infectious individuals or sources of infection?	Studies of population contact patterns or human-animal contact patterns (for zoonoses), information on types/categories of exposure	Detailed information on patterns of contact and other relevant exposures may not be available
	What is the availability and effectiveness of exposure-reduction measures (e.g., personal protective equipment)?	Epidemiological and other scientific studies of the effectiveness of exposure-reduction measures, information on adherence to and appropriate use of exposure-reduction measures	Scientific evidence may be limited, inconclusive or associated with high levels of uncertainty
Susceptibility to infection	Is exposure likely to lead to infection?	Mode(s) of transmission, infectious dose, per-contact transmission probability, epidemiological studies of transmission in household and other contacts	The relative contribution of different routes of infection to transmission may be unclear, the infectious dose may not be well established, the probability of infection from different types of exposure may not be known
	Are there factors that influence the likelihood of infection in exposed individuals?	Epidemiological studies of risk factors for infection, studies of infection risk among exposed individuals with medical or other vulnerabilities, data on vaccine effectiveness in relevant population groups (for vaccine-preventable diseases), pathogen mutations/adaptations	Scientific evidence for the risk of infection in specific population sub-groups may be limited, the extent to which vaccination prevents infection (rather than disease) may be unclear, relevant pathogen mutations may not be well characterized
Likely spread scenario(s)	What would be the likely extent of transmission over the period of assessment?	Mode(s) of transmission; reproduction number (*R*); serial interval and epidemic doubling time; population contact patterns (including human-animal contacts for zoonoses); transmission patterns in specific population sub-groups or occupational/exposure groups; availability and effectiveness of public health measures and medical countermeasures, including vaccines and antimicrobials; seasonal factors influencing transmission patterns; experience from similar events in the past	For novel pathogens, data on relevant epidemiological parameters of transmissibility may be limited; the effectiveness of measures to control transmission may be unclear
Impact on directly-affected individuals	What would be the health consequences on infected individuals?	Information on case-to-infection, case-to-hospitalization and case-to-fatality ratios; epidemiological studies of risk factors for severe outcomes; information on the frequency of severe outcomes among infected individuals; availability and effectiveness of medical countermeasures; information on the frequency of long-term sequelae of infection and consequences of infection on well-being	For novel pathogens, illness severity and case fatality may be over-estimated early on if detection is biased towards severe cases; the frequency and impact of long-term sequelae may be unclear; risk factors for severe illness may not be well established
	What additional consequences could there be for infected individuals?	Evidence of financial or other impacts on affected individuals and their families, including stigma and discrimination; information on additional burdens on affected individuals and families resulting from control measures	Financial and other impacts may be dependent on individual circumstances
Population impact	What fraction of the population would be affected? Would large numbers of severe cases and deaths be expected? Would health impacts affect the general population or be restricted to specific sub-groups?	Disease incidence, hospitalization, mortality, speed and geographic extent of epidemic spread, population sub-groups affected, long-term consequences of infection	The scale of health impacts may be highly dependent on uncertainties in the most likely spread scenario
	Would there be impacts to the health system and/or wider society?	Impacts on the health system, including healthcare workforce; societal disruption and economic impacts resulting from epidemic and/or associated control measures; public anxiety, social unrest and discrimination resulting from epidemic and/or associated control measures; impacts of epidemic and/or associated control measures on health inequalities	The range and extent of indirect impacts may be difficult to predict
